# Economic evaluation of cyclin-dependent kinases 4 and 6 inhibitors in advanced hormonal receptor-positive and human epidermal growth factor receptor 2 negative breast cancer: a nationwide budget impact analysis

**DOI:** 10.1080/20523211.2026.2626640

**Published:** 2026-02-19

**Authors:** Shereen Elazzazy, Nour Hisham Al-Ziftawi, Laila Shafei, Mohamed Izham Mohamed Ibrahim, Salha Bojassoum, Anas Hamad

**Affiliations:** a Pharmacy Department, The National Center for Cancer Care and Research, Hamad Medical Corporation, Doha, Qatar; b College of Pharmacy, QU Health, Qatar University, Doha, Qatar; c Medical Oncology and Palliative Care Medicine, The National Center for Cancer Care and Research, Hamad Medical Corporation, Doha, Qatar

**Keywords:** Health technology assessment, Cyclin-dependent kinase inhibitors, Hormone receptor-positive breast cancer, Resource allocation

## Abstract

**Background:**

Several trials demonstrated improvements in clinical outcomes associated with cyclin-dependent kinase 4 and 6 (CDK4/6) inhibitors in breast cancer patients. The challenge remains regarding their high costs. Ribociclib and Abemaciclib are cost-effective in Qatar. Yet, their affordability was not studied. This budget impact analysis (BIA) is to assess the affordability of adopting CDK4/6 inhibitors in Qatar over five years duration (2024-2028).

**Methods:**

We ran a BIA to evaluate two scenarios: (1) Increasing abemaciclib's market share from 20% to 60%, replacing both palbociclib and ribociclib. (2) Assuming equal market share for both ribociclib and abemaciclib up to 80%, reducing palbociclib's share. The analysis considered treatment costs, patient population, and disease prevalence. All data were retrieved from the National Center for Cancer Care and Research, and costs were presented in Qatari Riyals (QAR). Sensitivity analyses were run to ensure the robustness of the conclusion. All results were compared to Qatar's budget threshold, which is QAR 453,822.

**Results:**

Based on a total of 173 patients using CDK4/6 inhibitors, increasing abemaciclib's market share to 60% yielded cumulative savings of QAR 14 million over five years, which is around QAR 14,613 per patient per year. However, equally increasing ribociclib's and abemaciclib's market share to 80% resulted in a modest budget increase, remaining within acceptable thresholds. Sensitivity analyses confirmed the robustness of these findings, showing that cost reductions and higher uptake rates further enhanced savings.

**Conclusion:**

Abemaciclib is a budget-saving option for HR+/HER2- breast cancer in Qatar, should it replace the market share by up to 60% over five years. In addition, ribociclib and abemaciclib are affordable treatment options if they equally contributes to up to 80% of the market share for the eligible advanced breast cancer patients. The results supported the concept of allocating CDK4/6 inhibitors as they were found to be affordable to the Qatari healthcare system.

## 1. Background

Worldwide, breast cancer was indicated to be one of the most prevalent solid malignancies, specifically, patients who are hormone receptor-positive (HR+) and human epidermal growth factor receptor 2-negative (HER2-) were found to account for the majority of breast cancer patients. Yet, cyclin-dependent kinase 4 and 6 (CDK4/6) inhibitors, such as palbociclib (PLBO), ribociclib (RIBO), and abemaciclib (ABMA) has shown significant advantages in both progression-free survival (PFS) and overall survival (OS) in clinical trials (Finn et al., 2016; Hortobagyi et al., 2016; Goetz et al., 2017). The three approved CDK4/6 inhibitors differ in their dosing schedules, safety profiles, and monitoring requirements. Palbociclib and ribociclib are administered on a 21-day schedule followed by 7 days off the medication every 28 days, whereas abemaciclib is dosed continuously without a break (Finn et al., 2016; Hortobagyi et al., 2016). In terms of safety, neutropenia is more common with palbociclib and ribociclib, requiring frequent haematologic monitoring, while abemaciclib is associated with higher rates of gastrointestinal toxicity (Sledge et al., 2017). Nonetheless, their costs have formed a financial burden on the healthcare system and raised challenges in affordability and accessibility.

Several economic evaluations are considered vital to allow for assessing the economic impact of such advanced interventions and therapies. These economic evaluations include both cost-effectiveness analysis (CEA) and budget impact analysis (BIA) to inform decisions regarding the adoption of CDK4/6 inhibitors into clinical practice (Drummond et al., 2015). BIA offers multiple purposes compared to other economic evaluations. For example, CEA and cost-utility analysis (CUA) focus on efficiency – whether an intervention is cost-effective compared to other options based on effectiveness per patient (Drummond et al., 2015). In contrast, BIA examines whether the intervention is financially sustainable within a given budget (Drummond et al., 2015; Mauskopf et al., 2007). This means that if the budget impact of the new drug exceeds available resources, it may not be adopted even if it is cost-effective (Mauskopf et al., 2007; Sullivan et al., 2014). Other HTA components, such as cost–benefit analysis (CBA) and multi-criteria decision analysis (MCDA), evaluate broader societal perspectives and non-health outcomes (Thokala et al., 2016). Multiple HTAs should be considered when integrating high-cost interventions like CDK4/6 inhibitors for the sustainable utilisation of resources.

Comparative HTAs have assessed the relative value of individual CDK4/6 inhibitors. While some studies demonstrated a cost-effectiveness or cost-saving advantage for CDK4/6 inhibitors in the context of HR+/HER2 – advanced breast cancer (Galve-Calvo et al., 2018; Mistry, May, et al., 2018; Suri et al., 2019), the pooled results from a recent meta-analysis of cost-effectiveness analyses did not show any cost-effectiveness of using CDK4/6 inhibitors (Masurkar et al., 2024). In contrast, budget impact analyses have been conducted less extensively. For example, a BIA analysis examined the costs associated with implementing ribociclib plus letrozole over a 3-year period for eligible patients, revealing that ribociclib plus letrozole led to cumulative budget savings of $ 3.01 million per treated member per month in the United States (Mistry, Suri, et al., 2018).

Healthcare delivery in the Middle East and North Africa (MENA) region faces numerous challenges, including limited resources, disparities in healthcare infrastructure, and an increasing cancer burden (Katoue et al., 2022). Health Technology Assessments (HTAs) of CDK4/6 inhibitors in this region are crucial for ensuring adherence to innovative therapies and maintaining the sustainability of healthcare systems (Fasseeh et al., 2020). The outcome of this economic evaluation regarding pharmacotherapeutic regimens in breast cancer care settings remains inconclusive (Al-Ziftawi et al., 2021). Since Qatar is considered a high-income country in the MENA region, it has made significant progress in enhancing cancer management through multiple investment therapies. Nevertheless, integrating CDK4/6 inhibitors requires a comprehensive evaluation due to their huge financial implications. Limited data exist on the economic outcomes of CDK4/6 inhibitors in Qatar, highlighting the need for a thorough economic assessment. An initial exploratory analysis of RIBO versus PALBO in the first-line treatment of HR + advanced breast cancer indicated better outcomes with RIBO, suggesting a clinical difference between the two agents (Al-Ziftawi et al., 2022). However, when compared to ABMA, ABMA proved to be a cost-saving option relative to RIBO and PALBO (Elazzazy et al., 2024). These clinical distinctions between the three CDK4/6 agents may impose some economic implications. In NCCCR, abemaciclib (ABMA) could be less expensive, as continuous dosing without mandated cycle breaks reduces clinic visits and monitoring frequency, and the management of gastrointestinal adverse events is typically less resource-intensive than repeated blood count monitoring or hospitalisation for severe neutropenia (Elazzazy et al., 2024). Yet, the affordability and financial impact of ABMA and RIBO remain unclear. Conducting a BIA is considered an add on to existing cost-effectiveness studies to quantify the impact of CDK4/6 inhibitors on resources consumed in the healthcare system of Qatar.

This study aims to provide a budget impact analysis of CDK4/6 inhibitors in Qatar. It will model the economic implications of introducing these therapies into the Qatari healthcare system over a five-year period (2024-2028). Through evidence-based insights, this study intends to inform policymakers and healthcare providers about the cost-effectiveness and affordability of CDK4/6 inhibitors in Qatar, specifically ABMA and RIBO.

## 2. Methods

### 2.1. Models’ structures and validation

Two global budget impact models (BIM) were conducted to estimate the financial impact using TreeAge Pro software and implemented in Microsoft Excel 2010. Both models were adapted to suit the Qatari healthcare system and were assessed from the governmental perspective based on the main cancer care provider in Qatar, the National Center for Cancer Care and Research (NCCCR). Data on disease prevalence, treatment costs, and other relevant factors were modelled to determine the potential budget impact over a five-year period (2024–2028). Model outputs were reported annually as cost per treatment (for all patients) per year in Qatari Riyals (QAR) over five years. In accordance with the recommendations of the International Society for Health Economics and Outcomes Research (ISPOR) Task Force for reporting budget impact analysis in health (Sullivan et al., 2014), this analysis did not apply any discounting, inflation adjustments, or other cost adjustments over time.

The first model assumed that abemaciclib only had a partial market share versus the projected scenario where ABMA replaced both PLBO and RIBO sequentially, reaching up to 60%. The actual market component sizes for each scenario in the years of interest are as follows: 2024, 10%; 2025, 20%; 2026, 35%; 2027, 50%; and 2028, 60%. In contrast, the second model considered two scenarios: the current scenario with a limited share of ABMA and a major share of RIBO and PLBO versus the projected scenario of both ABMA and RIBO acquiring an equal share sequentially, reaching up to 80% while limiting any other options to 20%. The share would initially equal roughly 40 percent of the combinations – 40% in 2024, 50% in 2025, 60% in 2026, 70% in 2027, and 80% in 2028. The oncologists and clinical pharmacy experts on the team deemed these scenarios valid and realistic.

Both models were validated according to the AdViSHE framework to ensure accuracy and reliability (18). Conceptual validation and input validation were performed to confirm the model’s alignment with current clinical and market practices. Additionally, computerised model validation was conducted multiple times before adoption from TreeAge Pro software, using traces of various potential patient pathways from literature to guarantee reliability. Operational validation included expert assessment of model outcomes, cross-validation with similar studies, sensitivity analysis using alternative inputs, and comparison with empirical data.

### 2.2. Target population

The target population for analysis comprised adult women with HR+/HER2 – advanced or metastatic breast cancer who have not previously received chemotherapy for metastatic disease and are suitable candidates for endocrine therapy, as well as those who are not currently on CDK4/6 inhibitors. In the two scenario analyses, we evaluated patients with advanced or metastatic breast cancer who are candidates for treatments with ABMA, RIBO, or PALBO. According to the 2023 registry of patients, this included 173 current patients with a net expected prevalence increase of 5% based on the research team’s expert opinions.

### 2.3. Intervention & comparator

**Figure 1.**
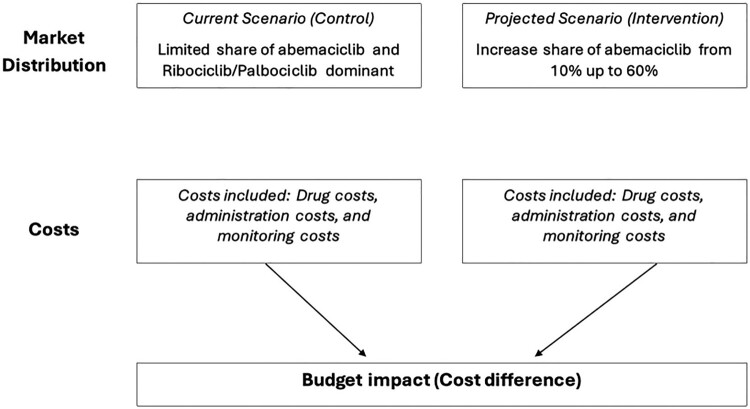
Budget impact analysis scheme (Scenario 1).

**Figure 2.**
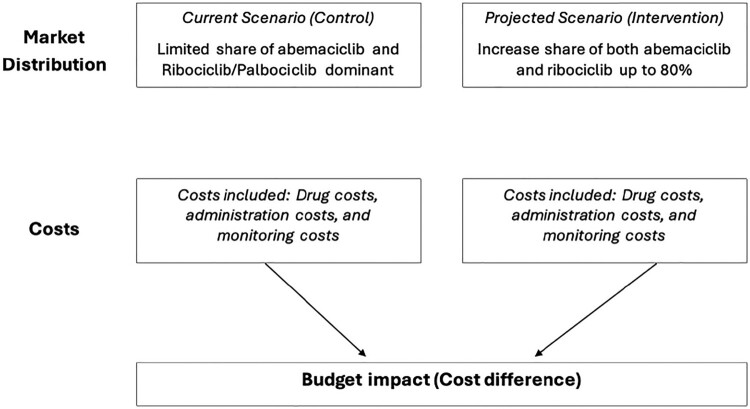
Budget impact analysis scheme (Scenario 2).

As mentioned in 2.1., two budget impact models will be run to cover the potential treatment trends in Qatar, a schematic diagram for each scenario is present in Figures 1 and 2.

*The first model:*

- **Current Scenario (Control)** – Abemaciclib maintains a limited market share while ribociclib and palbociclib dominate.
- **Projected Scenario (Intervention)** – gradually shifting abemaciclib to replace both palbociclib and ribociclib, reaching a **60% market share by 2028**, with market shares of 10%, 20%, 35%, 50%, and 60% in 2024, 2025, 2026, 2027, and 2028, respectively.

*The second model:*

- **Current Scenario (Control)** – Abemaciclib maintains a limited share while ribociclib and palbociclib dominate.
- **Projected Scenario (Intervention)** – Expansion of both abemaciclib and ribociclib’s combined market share, reaching **80% by 2028**, with annual increases from 40% in 2024 to 80% in 2028, while other treatment options, including palbociclib, gradually decrease to 20%.

### 2.4. Costs

Costs were estimated from the perspective of the NCCCR without discounting. These expenditures were directly obtained from the Department of Finance of Hamad Medical Corporation, to which the NCCCR belongs. Outcomes included the acquisition costs of CDK4/6 inhibitors, costs for combination drugs, laboratory examinations throughout the entire treatment period (complete blood count, blood chemistry tests, endocrinology tests, tumour markers, and coagulation tests), clinical radiology tests (X-ray, ultrasound, mammogram, MRI, CT, PET scan, and bone density DEXA scan), cardiac-related procedures for CDK4/6 inhibitors (ECG and echocardiogram), expenses for outpatient visits, and hospitalisation costs. A bottom-up costing approach comprising estimates of individual cost components was used to calculate costs, which were then summed to provide a total cost per scenario. All costs are expressed in QAR (USD 1 = QAR 3.65). Detailed cost data were similar to those used in our previous cost-effectiveness analysis (Elazzazy et al., 2024). Cost of medication per cycle and per year are present in Supplemental Material – C.

### 2.5 Sensitivity analysis

We did not conduct a probabilistic sensitivity analysis (PSA) as the BIA is consistent with ISPOR BIA guidelines. BIAs are typically deterministic in nature, focusing on one-way or scenario sensitivity analyses to capture uncertainty. Therefore, only one-way sensitivity analysis was conducted. A PSA was not conducted.

## 3. Results

### 3.1. Target population

The number of patients diagnosed with ER+/HER2 advanced breast cancer treated at NCCCR in Qatar reached a total of 173 in 2023. According to our medical records, we identified the total number of patients on each CKD4/6 inhibitor, with 30, 56, and 53 patients on ABMA, PLBO, and RIBO, respectively. Based on the 2023 prevalence data for Qatar, the current market distribution ratio is 33%:30%:18% for PLBO: RIBO: ABMA. We estimate the annual incidence of ER+/HER2 – ABC at 10%, with an expected 5% dropout rate each year, as suggested by our research team expert and based on last year’s epidemiologic estimates. Therefore, there is an annual net increase of 5% in the yearly available cases. Estimation was made assuming that patients are receiving CDK4/6 inhibitors yearly for 5 years.

### 3.2. First scenario: The abemaciclib market share increased over palbociclib and ribociclib by up to 60%

#### 3.2.1. Budget impact analysis results

**Figure 3.**
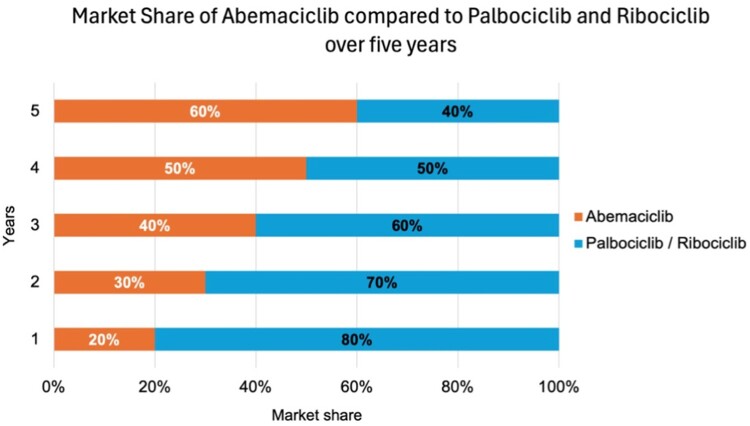
Bar chart representing the market share of Abemaciclib compared to Palbociclib and Ribociclib over the next five years, as the figure illustrates the increase of Abemaciclib’s distribution in the market share.

The starting point for speculating on the shares is that, at the end of year 1 in 2024 (with the current speculation adjustment), approximately 80% of the first line of advanced HR+/HER2 – breast cancer patients were treated with PLBO/RIBO plus endocrine treatment, and 20% with ABMA (as displayed in Figure 3). It was evident from the data that this resulted in savings of up to QAR 1,200,000 in the best-case scenario compared to the current scenario costs. Similar outcomes were observed in years 2 and 3, increasing the market share of ABMA by 30% and 40%, respectively. These results led to significant budget savings with incremental costs of QAR 1,900,000 and QAR 2,700,000. Furthermore, from years 4–5 of the project, by maintaining a 60% ABM intake ratio, we have achieved savings when comparing overall values to the current scenario. Even with the price increase over the five years, the total budget impact was less than QAR 14,000,000 in savings over that period. Table 1 provides a breakdown of the BIA results for ABMA market distribution.

**Table 1.** BIA of Abemaciclib replacing both Palbociclib and Ribociclib and standard care scenario until 60% uptake.

Cost based on current scenario (QAR)	Market share scenario	Market share scenario cost (QAR)	Incremental cost (budget impact) (QAR)^a^	Cost per patient (QAR)
**Year 1–2024 (N** **=** **173)**				
31,124,763	**Total**	**29,890,060**	−1,234,703	−7,137
	PLBO /RIBO/ Other TX 80%	24,899,810		
	ABMA 20%	4,990,249		
**Year 2–2025 (N** **=** **182)**				
32,681,001	**Total**	**30,736,344**	−1,944,658	−10,685
	PLBO /RIBO/ Other TX 70%	22,876,701		
	ABMA 30%	7,859,643		
**Year 3–2026 (N** **=** **191)**				
34,363,178	**Total**	**31,636,839**	−2,726,339	−14,274
	PLBO /RIBO/ Other TX 60%	20,617,907		
	ABMA 40%	11,018,932		
**Year 4–2027 (N** **=** **201)**				
36,081,336	**Total**	**32,503,017**	−3,578,320	−17,803
	PLBO/RIBO/ Other TX 50%	18,040,668		
	ABMA 50%	14,462,348		
**Year 5–2028 (N** **=** **211)**				
37,961,416	**Total**	**33,443,687**	−4,517,729	−21,411
	PLBO/RIBO/ Other TX 40%	15,184,566		
	ABMA 60%	18,259,121		
**Total budget impact over the years**		−14,001,749		

Abbreviations: PLBO; palbociclib, RIBO; ribociclib, ABMA; abemaciclib, Tx; standard treatment; QAR; Qatari riyals, N; number of patients per year.

^a^ Incremental cost was calculated by subtracting the total of ‘market share scenario cost’ from total of ‘Cost based on current scenario’.

#### 3.2.2. Sensitivity analysis results

The sensitivity analysis was undertaken to evaluate various parameters incorporated within the BIA. The parameters included the following: both standard care and ABMA prices and ABMA uptake ratio. detailed breakdown for the one-way sensitivity analysis is provided in Supplemental Material – A. The results were as follows:

I. Standard Care Cost Adjustments:

Simulation of sensitivity analysis of the cost of standard care changing by 10% to 25% has shown that in most cases permanent savings of the budget would be obtained. Year 1: A 10% reduction in standard care cost led to QAR 731,417 total savings. Over the 5 years, similar observation was seen, and the amount saved in Year 5 was QAR 2,676,226. But, when it was reduced by 25% the incremental cost was not cost saving, yet it was affordable. Budget savings in years 1–5, were between QAR 23,512 and QAR 86,030.

II. Abemaciclib Cost Adjustments:

Reducing ABMA’ s cost from 10% to up to 25% contributed to substantial budget savings across all five years. A 10% drop in budget savings of QAR 1,632,720 in Year 1, increasing annually, and reaching QAR 5,974,056 in Year 5. This trend was even more pronounced with the 25% reduction scenarios, yielding a budget-saving impact of up to QAR 8,158,546 in Year 5.

III. Cost Adjustments of both Standard Care and Abemaciclib:

The analysis indicated budget savings across a range of cost reductions from 10% to 25%. In year 1, a 10% reduction in treatments resulted in savings of QAR 1,129,434, then it was found to increase to QAR 4,132,552 in year 5. similar trend was observed with a 25% reduction. Across all scenarios, these results remained budget-saving.

IV. Abemaciclib Uptake Ratios:

Varying the ABMA uptake ratio by ±10% maintains the budget-saving outcome. When the uptake ratio increases by 10%, and the budget impact grows more favourably, reaching QAR 5,270,684 by year 5. Conversely, a 10% decrease also reflects savings, although slightly lower, with year 5 savings at QAR 3,764,774. This trend supports the robustness of ABMA adoption as a cost-saving measure within the specified range.

**Figure 4.**
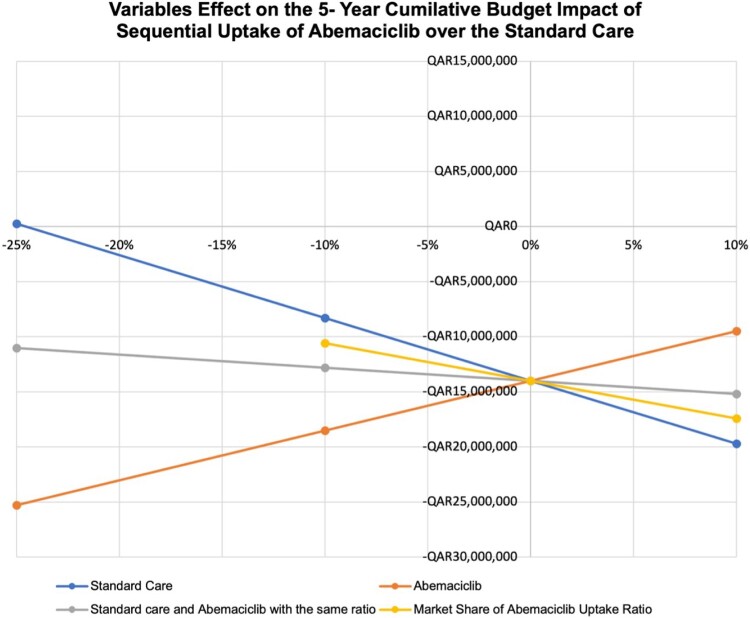
Multivariate sensitivity analysis represented by a spider plot for the effects of potential changes in prices across several data inputs. The steeper the slope of each factor, the greater the effect it has on the 5-year cumulative BIA.

In Figure 4, an illustration of the one-way sensitivity analyses represented by The Spider’s Plot shows the effects of possible changes in prices for ‘standard care’, ‘Abemaciclib’, and ‘both’, as well as the market share ratio on the budget impact of ABMA replacing the current standard care in the formulary. All parameters, including reductions in cost and an increase in the ABMA uptake ratio, led to substantial budget savings. The most significant savings were achieved by changing the costs of both standard care and ABMA. The figure underscores the potential financial benefit of adopting ABMA as a substitute for PLBO and RIBO plus standard care.

### 3.3. Second scenario: abemaciclib and ribociclib equally taken up over palbociclib up to 80% of the current market

#### 3.3.1. Budget impact analysis results

**Figure 5.**
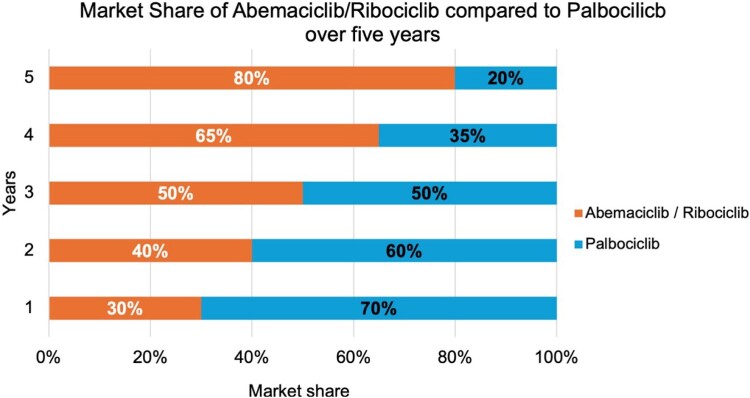
Chart representing the market share of Abemaciclib and Ribociclib compared to Palbociclib over the next five years, as the figure illustrates the increase in the distribution of Abemaciclib and Ribociclib’s market share.

In year 1, it was assumed that PLBO and other standard treatments such as hormonal therapy or aromatase inhibitors accounted for about 70% of the first line treatment market share of HR+/HER2- advanced breast cancer, while ABMA and RIBO were evenly distributed over 30% of the market as shown in Figure 5. Findings revealed that the method was affordable, as compared to the current budget, it raises the current budget by QAR 4,568 per patient as opposed to Qatar's threshold of QAR 45,382 per patient. In years 2 and 3, similar trends of results were observed, which led to the increase in the market share of ABM and RIBO to 40% and 50% along the respective quarters. This has resulted in a significant cost-effective increase in the overall budget of QAR 6,079 and QAR 7,613 per patient as compared to [QAR 45,382] ±   QAR 5,677. Furthermore, over years 4 and 5, the incremental distribution of ABMA and RIBO has always been below Qatar’s budget impact threshold. Results of ASMA and RIBO BIA are equally divided in Table 2.

**Table 2.** BIA of Abemaciclib and Ribociclib is equally taken until making both 80% of the market scenario.

Cost based on current scenario (QAR)	Market share scenario	Market share scenario cost (qar)	Incremental cost (budget impact) (QAR)^a^	Cost per patient (QAR)
**Year 1–2024 (N** **=** **173)**				
27,102,920	**Total**	**27,893,201**	790,281	4,568
	PLBO/ Other Tx 70%	18,972,044		
	ABMA & RIBO 30%	8,921,157		
***Year 2–2025 (N*** ***=*** ***182)***				
28,458,066	**Total**	**29,564,460**	1,106,393	6,079
	PLBO/ Other Tx 60%	17,074,840		
	ABMA & RIBO 40%	12,489,620		
**Year 3–2026 (N** **=** **191)**				
29,922,877	**Total**	**31,377,055**	1,454,178	7,613
	PLBO/ Other Tx 50%	14,961,439		
	ABMA & RIBO 50%	16,415,617		
**Year 4–2027 (N** **=** **201)**				
31,419,021	**Total**	**33,403,974**	1,984,953	9,875
	PLBO/ Other Tx 35%	10,996,657		
	ABMA & RIBO 65%	22,407,317		
**Year 5–2028 (N** **=** **211)**				
33,056,163	**Total**	**35,626,479**	2,570,316	12,182
	PLBO/ Other Tx 20%	6,611,233		
	ABMA & RIBO 80%	29,015,247		

Abbreviations: PLBO; palbociclib, RIBO; ribociclib, ABMA; abemaciclib, Tx; standard treatment; QAR; Qatari riyals, N; number of patients per year.

^a^ Incremental cost was calculated by subtracting the total of ‘market share scenario cost’ from total of ‘Cost based on current scenario’.

#### 3.3.2. Sensitivity analysis

The sensitivity analysis was undertaken to evaluate various parameters incorporated within the BIA, which included the following: both standard care and ABMA, as well as RIBO prices and their uptake ratios. There is a detailed breakdown for the one-way sensitivity analysis in Supplemental Material – B. Results were as follows:

I. Standard Care Price:

Reducing costs by 10% to 25% resulted in an affordable increase in the total budget, ranging from QAR 1.4 million to QAR 7.7 million over five years. All assumptions led to an overall budget impact within an acceptable range, which did not exceed our indicated threshold of QAR 45,382. Therefore, it implied that moderate reductions in standard care prices do not substantially shift the budget.

II. Changing *Abemaciclib and Ribociclib Costs:*

When ABMA and RIBO costs were reduced by 10%, the budget impact was minimal, keeping it within the affordable range (below QAR 20000/year) annually. However, a 25% cost reduction resulted in budget savings, which saved up to QAR 3.4 million by year 5. Therefore, it was implied that substantial reductions in medication costs led to budget savings.

III. Cost Reduction of Standard Care, Abemaciclib, and Ribociclib:

Adjusting both standard care and ABMA/RIBO costs by 10% or 25% resulted in a consistent trend of affordability, with incremental costs mostly up to QAR 1.7 million per year. However, a mixed reduction of 10% for standard care and up to 25% for ABMA/RIBO led to budget savings over the five years, with incremental costs ranging between QAR 418,415 and QAR 1,360,858 from year 1 to year 5.

IV. Abemaciclib and Ribociclib Uptake Ratio:

Adjusting the uptake ratio by −10% or +10% resulted in incremental costs ranging from QAR 500,000 to QAR 2.9 million per year, while the budget impact remained manageable over five years. The findings indicated that changing uptake ratios has a moderate effect on the budget but did not lead to any unacceptable ranges.

**Figure 6.**
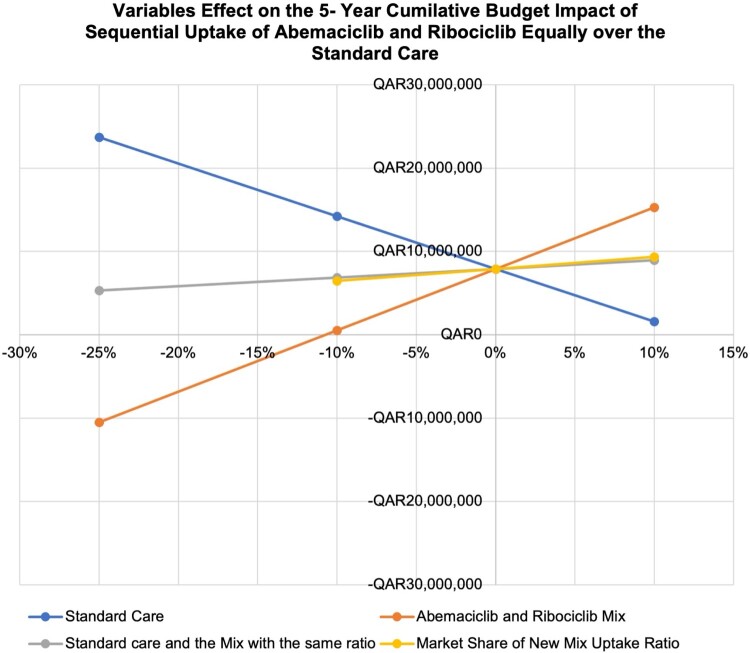
Multivariate sensitivity analysis represented by a spider plot for the effects of possible price changes on several input data. The steeper the slope of each factor, the greater its impact on the 5-year cumulative BIA.

The sensitivity analysis findings are summarised in the ‘Spider Diagram’ represented in Figure 6.

## 4. Discussion

Prior to this current BIA, a study was conducted to compare the cost-effectiveness of PALBO and RIBO in stage IV HR+/HER-2 negative breast cancer patients in Qatar. A 10-year Markov model was developed, considering all direct costs from the hospital perspective (Al-Ziftawi et al., 2022). The model included three health states: progression-free (PFS), progressed disease (PD), and death. The analysis revealed that PALBO resulted in a total of QAR 372,663 per patient, with an additional 71.62 life months (5.986 years), which translates to an overall QALY of 3.058 per patient. In contrast, RIBO’s estimated 10-year cost was QAR 333,584, with a gain of 75.96 months (6.33 years) and total QALYs of 3.160 (Al-Ziftawi et al., 2022). Our analysis indicated that RIBO is dominant over PALBO in terms of being more effective and less costly (Al-Ziftawi et al., 2022). Following this model, we developed another 10-year Markov model to compare all CDK4/6 inhibitors in combination with letrozole as a first-line treatment for HR+/HER-2 negative advanced breast cancer (Elazzazy et al., 2024). The results showed that RIBO was the most effective option, followed by PALBO and ABMA (Elazzazy et al., 2024), indicating that RIBO was dominant over PALBO but was not cost-effective compared to ABMA, with an ICER of 1,588,545 QAR/QALY (Elazzazy et al., 2024).

After conducting the previously mentioned cost-effectiveness models, it was deemed appropriate to perform a BIA to assess whether the use of CDK4/6 agents could provide a financially sustainable economic outcome (Smith & Levy, 2023 Sep 30; World Health et al., 2003). Since all three CDK4/6 agents are included in the HMC medication formulary, this analysis is intended to evaluate the affordability and economic impact of the three agents on the budget over the upcoming five years by modelling different scenarios that vary the medications’ uptake ratio over the years.

The WHO recommended adopting a threshold that is three times the GDP per capita (International Monetary Fund, 2025; Woods et al., 2016). However, a threshold of 1.5 times the GDP was adopted in our BIA, secondary to Qatar’s high-income level and GDP per capita. As per the International Monetary Fund (IMF), it was reported that Qatar’s GDP per capita in 2022 was QAR 302,172. The adopted threshold was, therefore, QAR ,822 per patient per year. An incremental budget impact cost larger than this threshold would be considered to have an unacceptable budget impact if it exceeded 10% of this threshold (Borrelli et al., 2021).

This paper conducted a BIA to assess two hypothetical scenarios concerning the use of CDK4/6 inhibitors at NCCCR in Qatar, based on what clinical experts identified as likely to be the case. Hence, it needed to evaluate the lowest-cost option, which was ABMA that replaces both RIBO and PLBO. Furthermore, assessing the second assessment scenario involved maintaining an equal distribution of ABMA and RIBO over the years, which seemed a more realistic situation, especially for those who have already started on RIBO, while guaranteeing the availability of more than one CDK4/6 agent within the formulary.

It was assumed, in the first scenario in this paper, that the market share of ABMA is expected to grow from 20% to 60% over five years. By replacing PLBO and RIBO with ABMA, the BIA revealed substantial budget savings with an expectation that a total QAR 2,700,000 will likely be saved over five years. Hence, the presence of ABMA primarily across the healthcare Qatari market provides considerable budgetary savings. Similar to our findings, Borreli et al performed a BIA modelling the addition of ABMA to treatment regimens from the perspectives of commercial and Medicare US payers (Borrelli et al., 2021). The study projected an increase in the overall per-patient costs, estimating a total of US$ 1,804,469 and US$ 6,588,572 from the commercial and Medicare perspectives, respectively (Borrelli et al., 2021). The difference between their findings and ours could be attributed to the significant difference between Qatar’s and the USA’s patient populations. In Qatar, the total population estimated was 173 patients with a prevalence of 10%; whereas, in the USA, the prevalence is almost 30% (Price et al., 2022). On the other hand, a BIA was carried out to evaluate the incremental cost-effectiveness of ABMA plus endocrine therapy from US commercial health plan perspective (Davidoff & Powe, 1996 Winter). ABMA also had an incremental budget impact of US$ 4,181,061 per year, so these offsets appear to be cumulative over time (Davidoff & Powe, 1996 Winter). In contrast, our analysis, raising the proportion of ABMA to 60% by the 5th year resulted in a considerably positive budget impact with a total cost saving of QAR 4,517,729 from the healthcare system point of system. The overall cost – saving benefit of ABMA compared to the US in our setting, from a payer perspective, likely reflects differences between payer and healthcare perspectives that impact WTP thresholds in health economics. Payers, such as insurance companies, are concerned with cost containment and the immediate financial impact of treatments, while the healthcare perspective is broader, incorporating societal benefits (e. g., long – term health outcomes and productivity gains) (Iino et al., 2022; Teptsova et al., 2018). The third – party payer perspective results in lower WTP thresholds since immediate and tangible value is recognised, but the healthcare perspective is more holistic and would justify higher expenditures when value occurs further in time (Avxentyev et al., 2018).

In the second scenario, it was assumed that the market share of both ABMA and RIBO is to increase equally, starting from 30% up to 80% over five years, replacing PLBO. While the BIA demonstrated an increase in total costs under this scenario, the increase was deemed affordable and within the acceptable budget impact range. This upward trend continued as ABMA and RIBO's combined market share rose by year 5. The undertaken sensitivity analysis indicated that the results were robust to the conclusion upon changing the costs of medications and the uptake ratio of ABMA and RIBO. Moreover, the analysis demonstrated greater savings when the costs of ABMA and RIBO were reduced, and the ratio of uptake was increased.

A previous BIA evaluated the use of both RIBO and PLBO as first-line treatment for HR-positive HER2-negative advanced breast cancer from the Russian healthcare perspective. The study emphasised that RIBO drove lower costs compared to PLBO by a US$18,653 difference. Replacing PLBO with RIBO over five years led to savings of US$ 322 million (Zhu et al., 2022). This cost reduction was attributed to dose reductions, which made RIBO more cost-effective (Zhu et al., 2022). Moreover, Zhu et al highlighted lower drug wastage costs with RIBO compared to PLBO due to different dosing patterns and the availability of RIBO in a single strength (Djambazov et al., 2018). On the contrary, if the market share of ABMA and RIBO were to increase over PLBO, a positive budget impact would be seen, resulting in savings of approximately QAR 2 million.

Nevertheless, a study conducted in Bulgaria investigated the impact of the addition of PLBO to endocrine therapy and increasing the market share over 5 years (Mistry, Suri, et al., 2018). The BIA indicated that the incorporation would lead to saving up to 657,047 BGN (USD 348,768) during the first year. Consequently, by reaching the fifth year, the assumed scenario led to a total saving of 11,167,298 BGN (USD 59,277,24) (Mistry, Suri, et al., 2018). In contrast, our analysis did not evaluate PBLO’s market share increase. In fact, our results highlighted the incremental budget impact of QAR 790,281 in the 1^st^ year, reaching up to QAR 2,570,316 by the 5th year, secondary to increasing the market share of both ABMA and RIBO equally over PLBO. Such discrepancy could be secondary to differences in ADEs rates or pricing plans of PLBO in Bulgaria. A BIA conducted in the US by Mistry et al., to assess the economic impact of adding RIBO to endocrine therapy as a 1st line treatment for HR + ve/HER-2 negative BC patients (Møller & Oddershede, 2019). This analysis, conducted from the US payer perspective, revealed a total saving of USD 3.01 million (Møller & Oddershede, 2019). In alignment with our analysis, increasing the market share of ABMA/RIBO over PLBO led to affordable costs within the acceptable budget impact threshold. It was further proven by Møller et al. in their recent BIA conducted in Denmark that RIBO was associated with a total saving of approximately DKK 4.3 million (USD 597,926) over two years (Møller & Oddershede, 2019). Previous studies reported lower costs attributed to RIBO compared to PALBO, due to having a lower linear acquisition cost for smaller doses, as RIBO has a flat acquisition cost for all three doses (Suri et al., 2019). On the contrary, our analysis evaluated the distribution of both ABMA and RIBO equally over PLBO; therefore, our results could not be attributed solely to RIBO.

This paper aimed to have some implications on the Qatari healthcare system. In our economic analysis, RIBO was the most effective CDK inhibitor and dominant over PLBO, but not cost-effective compared to ABMA. While PLBO failed to be cost-effective against both ABMA and RIBO (Elazzazy et al., 2024), ABMA was the most cost-efficient agent, though not the most effective (Elazzazy et al., 2024). From this standpoint, our current BIA further confirms cost-saving outcomes by increasing the market share of ABMA over PLBO and RIBO in the upcoming 5 years. Additionally, it was confirmed that increasing the market share of both ABMA and RIBO equally still imposes an increase in costs that remain within the acceptable budget impact threshold. It is important to note that the occurrence of treatment-related adverse events has important economic implications for healthcare systems. As the management of these adverse events lead to increased healthcare resource utilisation and, consequently, higher overall treatment costs. Therefore, to mitigate the financial uncertainty associated with adverse-event-related expenditures, policy measures could be considered.

This BIA is believed to have several strengths. Firstly, this is the first BIA analysis evaluating the use of CKD4/6 inhibitors in Qatar and the Gulf area. Also, this study assessed multiple proposed scenarios over a five-year period, providing an understanding of the different clinical practice approaches that can be adopted with CDK4/6 inhibitors. On the other hand, one of those limitations could impact the study. For example, it was assumed that the market share of both ABMA and RIBO would increase in a fixed trajectory, which might not entirely reflect real clinical practice. Additionally, the inability to publish the cost per unit could lead to limitations in the reproducibility of this model.

Several pathways can be proposed as future research ideas; for instance, future studies could investigate different pricing strategies for PBLO, considering that it is the costliest CDK4/6 agent. Also, it is worth considering conducting a BIA to assess the combination therapy of CDK4/6 inhibitors with oestrogen receptor antagonists or other immunotherapy agents in breast cancer. Furthermore, a BIA could be conducted comparing RIBO to PLBO over a five-year horizon, excluding ABMA, since previous research indicated positive results with RIBO, noting that it demonstrated cost-effective results compared to PLBO (Elazzazy et al., 2024). While our results suggest that ABMA is associated with potential budget savings, it is important to highlight that these estimates remain sensitive to drug pricing and real-world patterns of monitoring. From a policy perspective, the next steps should focus on negotiating affordable pricing agreements and closely tracking real-world utilisation and costs to ensure that the projected savings are realised in practice and to maintain financial sustainability for the healthcare system.

## 5. Conclusion

In this paper, we report the five-year budget impact in Qatar of treatment with HR-positive, HER2-negative advanced breast cancer using CDK4/6 inhibitors and using ABMA plus endocrine therapy exclusively up to 80% of the current treatment options displayed marked cost reductions, estimating a 14,001,749 QAR (3,836,095 USD) reduction from baseline by year five, indicating that ABMA is superior in budget-saving compared to both PLBO and RIBO. Should both ABMA and RIBO have equal market share, this would result in a slight cost increase; however, this is a more realistic scenario that provides healthcare practitioners. Under the stated assumptions and current prices, ABMA appears to be budget-saving; however, the findings remain sensitive to both pricing and market uptake. Generated conclusions were based on a budget impact threshold of 1.5 GDP per capita. Future considerations should involve supply chain price negotiations, ongoing budget monitoring, and further analyses incorporating PSA. The findings of this analysis would be significant for healthcare policymakers and decision-makers in Qatar.

## Supplementary Material

Supplemental Material

## Data Availability

The data presented in this study are available on request from the corresponding author.
