# Supplementary material for: Economic evaluation of cyclin-dependent kinases 4 and 6 inhibitors in advanced hormonal receptor-positive and human epidermal growth factor receptor 2 negative breast cancer: a nationwide budget impact analysis
Source: J Pharm Policy Pract. 2026 Feb 19;19(1):2626640. doi: 10.1080/20523211.2026.2626640 (PMC12922424; doi:10.1080/20523211.2026.2626640)
Supplement: Supplemental Material [file JPPP_A_2626640_SM6426.docx]

**Supplementary Material**

**Supplementary Material – A**

Breakdown of the one-way sensitivity analysis conducted on the first scenario: Abemaciclib market share increase over palbociclib and ribociclib up to 60%

| **Parameter** | **Uncertainty Range** | **Year** | **Current Scenario Cost (QAR)** | **Market Share Scenario Cost (QAR)** | **Budget Impact (QAR)** | **Impact on base case conclusion** |
| --- | --- | --- | --- | --- | --- | --- |
| **Standard Care Price** | - 10% | **Year 1** | 28,608,333 | 27,876,915 | - 731,417 | Budget-saving |
|  | - 25% |  | 24,833,678 | 24,857,199 | 23,512 | **Change from base case (Affordable)** |
|  | - 10% | **Year 2** | 30,038,749 | 28,886,767 | - 1,151,892 | Budget-saving |
|  | - 25% |  | 26,075,371 | 26,112,403 | 37,031 | **Change from base case (Affordable)** |
|  | - 10% | **Year 3** | 31,584,922 | 29,969,885 | - 1,615,037 | Budget-saving |
|  | - 25% |  | 28,788,416 | 27,469,455 | 51,917 | **Change from base case (Affordable)** |
|  | - 10% | **Year 4** | 33,164,168 | 31,044,433 | - 2,119,736 | Budget-saving |
|  | - 25% |  | 30,288,485 | 28,856,556 | 68,141 | **Change from base case (Affordable**) |
|  | - 10% | **Year 5** | 34,892,244 | 32,216,018 | - 2,676,226 | Budget-saving |
|  | - 25% |  | 30,288,485 | 30,374,515 | 86,030 | **Change from base case (Affordable)** |
| **Abemaciclib Price** | - 10% | **Year 1** | 31,124,763 | 29,492,043 | - 1,632,720 | Budget-saving |
|  | - 25% |  | 31,124,763 | 28,895,018 | - 2,229,745 | Budget-saving |
|  | - 10% | **Year 2** | 32,681,001 | 30,109,467 | - 2,571,534 | Budget-saving |
|  | - 25% |  | 32,681,001 | 29,169,153 | - 3,511,848 | Budget-saving |
|  | - 10% | **Year 3** | 34,363,178 | 30,757,981 | - 3,605,196 | Budget-saving |
|  | - 25% |  | 34,363,178 | 29,439,695 | - 4,923,483 | Budget-saving |
|  | - 10% | **Year 4** | 36,081,336 | 31,349,516 | - 4,731,820 | Budget-saving |
|  | - 25% |  | 36,081,336 | 29,619,266 | - 6,462,071 | Budget-saving |
|  | - 10% | **Year 5** | 37,961,416 | 31,987,360 | - 5,974,056 | Budget-saving |
|  | - 25% |  | 37,961,416 | 29,802,870 | - 8,158,546 | Budget-saving |
| **Changing the price of both (standard : Abemaciclib)** | - 10% : - 10% | **Year 1** | 28,608,333 | 27,478,899 | - 1,129,434 | Budget-saving |
|  | - 10% : - 25% |  | 28,608,333 | 26,881,874 | - 1,726,459 | Budget-saving |
|  | - 25% : - 10% |  | 24,833,687 | 24,459,182 | - 374,505 | Budget-saving |
|  | - 25% : -25% |  | 24,833,687 | 23,862,157 | - 971,529 | Budget-saving |
|  | - 10% : - 10% | **Year 2** | 30,038,749 | 28,259,891 | - 1,778,858 | Budget-saving |
|  | - 10% : - 25% |  | 30,038,749 | 27,319,577 | - 2,719,172 | Budget-saving |
|  | - 25% : - 10% |  | 26,075,371 | 25,485,526 | - 589,845 | Budget-saving |
|  | - 25% : -25% |  | 26,075,371 | 24,454,212 | - 1,530,159 | Budget-saving |
|  | - 10% : - 10% | **Year 3** | 31,584,922 | 29,091,028 | - 2,493,894 | Budget-saving |
|  | - 10% : - 25% |  | 31,584,922 | 27,772,742 | - 3,812,180 | Budget-saving |
|  | - 25% : - 10% |  | 27,417,539 | 26,590,598 | - 826,941 | Budget-saving |
|  | - 25% : -25% |  | 27,417,539 | 25.972,805 | - 2,145,227 | Budget-saving |
|  | - 10% : - 10% | **Year 4** | 33,164,168 | 29,890,932 | - 3,273,236 | Budget-saving |
|  | - 10% : - 25% |  | 33,164,168 | 28,160,681 | - 5,003,487 | Budget-saving |
|  | - 25% : - 10% |  | 28,788,416 | 27,703,056 | - 1,085,360 | Budget-saving |
|  | - 25% : -25% |  | 28,788,416 | 26,972,805 | - 2,815,610 | Budget-saving |
|  | - 10% : - 10% | **Year 5** | 34,892,244 | 30,759,691 | - 4,132,552 | Budget-saving |
|  | - 10% : - 25% |  | 34,892,244 | 28,575,201 | - 6,317,042 | Budget-saving |
|  | - 25% : - 10% |  | 30,288,485 | 28,918,188 | - 1,370,297 | Budget-saving |
|  | - 25% : -25% |  | 30,288,485 | 26,733,698 | - 3,554,787 | Budget-saving |
| **Abemaciclib uptake ratio** | - 10% | **Year 1** | 31,124,763 | 30,507,411 | - 671,352 | Budget-saving |
|  | + 10% |  | 31,124,763 | 29,272,708 | - 1,852,055 | Budget-saving |
|  | - 10% | **Year 2** | 32,681,001 | 31,384,563 | - 1,296,438 | Budget-saving |
|  | +10% |  | 32,681,001 | 30,088,124 | - 2,592,877 | Budget-saving |
|  | - 10% | **Year 3** | 34,363,178 | 32,318,423 | - 2,044,754 | Budget-saving |
|  | +10% |  | 34,363,178 | 30,955,254 | - 3,407,924 | Budget-saving |
|  | - 10% | **Year 4** | 36,081,336 | 33,218,681 | - 2,862,656 | Budget-saving |
|  | +10% |  | 36,081,336 | 31,787,353 | - 4,293,984 | Budget-saving |
|  | - 10% | **Year 5** | 37,961,416 | 34,196,642 | - 3,764,774 | Budget-saving |
|  | +10% |  | 37,961,416 | 32,690,732 | - 5,270,684 | Budget-saving |

**Supplementary Material – B**

Breakdown of the one-way sensitivity analysis conducted on the second scenario: Abemaciclib and ribociclib Equally distributed over palbocicilib up to 80%

| **Parameter** | **Uncertainty Range** | **Year** | **Current Scenario Cost (QAR)** | **Market Share Scenario Cost (QAR)** | **Budget Impact (QAR)** | **Impact on base case conclusion** |
| --- | --- | --- | --- | --- | --- | --- |
| **Standard Care Price** | - 10% | **Year 1** | 24,994,781 | 26,417,504 | 1,422,723 | Affordable change |
|  | - 25% |  | 21,932,573 | 24,203,958 | 2,371,385 | Affordable change |
|  | - 10% | **Year 2** | 26,244,520 | 28,236,332 | 1,991,812 | Affordable change |
|  | - 25% |  | 22,924,202 | 26,244,141 | 3,319,939 | Affordable change |
|  | - 10% | **Year 3** | 27,595,395 | 30,213,314 | 2,617,919 | Affordable change |
|  | - 25% |  | 24,104,170 | 28,467,702 | 4,363,532 | Affordable change |
|  | - 10% | **Year 4** | 28,975,164 | 32,548,624 | 3,573,460 | Affordable change |
|  | - 25% |  | 25,309,379 | 31,265,599 | 5,956,220 | Affordable change |
|  | - 10% | **Year 5** | 30,484,965 | 35,112,240 | 4,627,275 | Affordable change |
|  | - 25% |  | 26,628,167 | 34,340,880 | 7,712,713 | Affordable change |
| **Abemaciclib and Ribociclib Prices** | - 10% | **Year 1** | 27,102,920 | 27,156,746 | 53,826 | Affordable change |
|  | - 25% |  | 27,102,920 | 26,052,063 | - 1,050,857 | **Change from base case (Budget-saving)** |
|  | - 10% | **Year 2** | 28,458,066 | 28,533,422 | 75,356 | Affordable change |
|  | - 25% |  | 28,458,066 | 26,986,867 | - 1,471,200 | **Change from base case (Budget-saving)** |
|  | - 10% | **Year 3** | 29,922,877 | 30,021,921 | 99,044 | Affordable change |
|  | - 25% |  | 29,922,877 | 27,989,220 | - 1,933,658 | **Change from base case (Budget-saving)** |
|  | - 10% | **Year 4** | 31,419,021 | 31,554,216 | 135,194 | Affordable change |
|  | - 25% |  | 31,419,021 | 28,779,578 | - 2,639,658 | **Change from base case (Budget-saving)** |
|  | - 10% | **Year 5** | 33,056,163 | 33,231,226 | 175,063 | Affordable change |
|  | - 25% |  | 33,056,163 | 29,638,347 | - 3,417,816 | **Change from base case (Budget-saving)** |
| **Changing the price of both (standard : Abemaciclib and Ribociclib)** | - 10% : - 10% | **Year 1** | 24,994,781 | 25,681,049 | 686,267 | Affordable change |
|  | - 10% : - 25% |  | 24,994,781 | 24,576,366 | - 418,415Q | **Change from base case (Budget-saving)** |
|  | - 25% : - 10% |  | 21,832,573 | 23,467,503 | 1,634,930 | Affordable change |
|  | - 25% : -25% |  | 21,832,573 | 22,362,820 | 530,247 | Affordable change |
|  | - 10% : - 10% | **Year 2** | 26,244,520 | 27,205,295 | 960,774 | Affordable change |
|  | - 10% : - 25% |  | 26,244,520 | 25,658,739 | - 585,781 | **Change from base case (Budget-saving)** |
|  | - 25% : - 10% |  | 22,924,202 | 25,213,104 | 2,288,902 | Affordable change |
|  | - 25% : -25% |  | 22,924,202 | 23,666,548 | 742,346 | Affordable change |
|  | - 10% : - 10% | **Year 3** | 27,595,395 | 28,858,179 | 1,262,785 | Affordable change |
|  | - 10% : - 25% |  | 27,595,395 | 26,825,478 | - 769,916 | **Change from base case (Budget-saving)** |
|  | - 25% : - 10% |  | 24,104,170 | 27,112,567 | 3,008,397 | Affordable change |
|  | - 25% : -25% |  | 24,104,170 | 25,079,866 | 975,696 | Affordable change |
|  | - 10% : - 10% | **Year 4** | 28,975,164 | 30,698,866 | 1,723,702 | Affordable change |
|  | - 10% : - 25% |  | 28,975,164 | 27,924,228 | - 1,050,936 | **Change from base case (Budget-saving)** |
|  | - 25% : - 10% |  | 25,309,379 | 29,415,841 | 4,106,462 | Affordable change |
|  | - 25% : -25% |  | 25,309,379 | 26,641,203 | 1,331,825 | Affordable change |
|  | - 10% : - 10% | **Year 5** | 30,484,965 | 32,716,987 | 2,232,022 | Affordable change |
|  | - 10% : - 25% |  | 30,484,965 | 29,124,107 | - 1,360,858 | **Change from base case (Budget-saving)** |
|  | - 25% : - 10% |  | 26,628,167 | 31,945,627 | 5,317,460 | Affordable change |
|  | - 25% : -25% |  | 26,628,167 | 28,352,474 | 1,724,581 | Affordable change |
| **Abemaciclib and Ribociclib uptake ratio** | - 10% | **Year 1** | 27,102,920 | 27,629,774 | 526,854 | Affordable change |
|  | + 10% |  | 27,102,920 | 28,156,628 | 1,053,708 | Affordable change |
|  | - 10% | **Year 2** | 28,458,066 | 29,287,861 | 829,795 | Affordable change |
|  | +10% |  | 28,458,066 | 29,841,058 | 1,382,992 | Affordable change |
|  | - 10% | **Year 3** | 29,922,877 | 31,086,220 | 1,163,342 | Affordable change |
|  | +10% |  | 29,922,877 | 31,667,891 | 1,745,013 | Affordable change |
|  | - 10% | **Year 4** | 31,419,021 | 33,098,597 | 1,679,575 | Affordable change |
|  | +10% |  | 31,419,021 | 33,709,351 | 2,290,330 | Affordable change |
|  | - 10% | **Year 5** | 33,056,163 | 35,305,190 | 2,249,027 | Affordable change |
|  | +10% |  | 33,056,163 | 35,947,769 | 2,891,606 | Affordable change |

**Supplementary Material – C**

Resource use table for the costs per cycle of CDK4/6 inhibitors included in the budget impact analysis

| **Medication** | **Cost of Medication Per Cycle Per Patient** | **Cost of Medication per year Per Patient** |
| --- | --- | --- |
| **Abemaciclib** | QAR 5,888 | QAR 70,656 |
| **Palbociclib** | QAR 15,498 | QAR 185,976 |
| **Ribociclib** | QAR 5,418 | QAR 65,016 |
